# Comparative Transcriptome Analysis Unveils Regulatory Factors Influencing Fatty Liver Development in Lion-Head Geese under High-Intake Feeding Compared to Normal Feeding

**DOI:** 10.3390/vetsci11080366

**Published:** 2024-08-11

**Authors:** Jie Kong, Ziqi Yao, Junpeng Chen, Qiqi Zhao, Tong Li, Mengyue Dong, Yuhang Bai, Yuanjia Liu, Zhenping Lin, Qingmei Xie, Xinheng Zhang

**Affiliations:** 1 State Key Laboratory of Swine and Poultry Breeding Industry & Heyuan Branch, Guangdong Provincial Laboratory of Lingnan Modern Agricultural Science and Technology, College of Animal Science, South China Agricultural University, Guangzhou 510642, China; dreamgirl0623@stu.scau.edu.cn (J.K.); ziqiy@stu.scau.edu.cn (Z.Y.); qiqi-zhao@stu.scau.edu.cn (Q.Z.); ngwjsm@stu.scau.edu.cn (T.L.); 20223139018@stu.scau.edu.cn (M.D.); 20223139001@stu.scau.edu.cn (Y.B.); 2 Guangdong Provincial Key Lab of AgroAnimal Genomics and Molecular Breeding, College of Animal Science, South China Agricultural University, Guangzhou 510642, China; 3 Guangdong Engineering Research Center for Vector Vaccine of Animal Virus, Guangzhou 510642, China; 4 Zhongshan Innovation Center, South China Agricultural University, Zhongshan 528400, China; 5 Shantou Baisha Research Institute of Original Species of Poultry and Stock, Shantou 515000, China; chenjunpeng02@scau.edu.cn (J.C.); linzp02@scau.edu.cn (Z.L.); 6 College of Coastal Agricultural Sciences, Guangdong Ocean University, Zhanjiang 524088, China; liuyuanjia@gdou.edu.cn

**Keywords:** lion-head goose, transcriptome, high intake, feeding, fatty liver

## Abstract

**Simple Summary:**

The study’s findings on differential gene expression in lion-head geese under high-energy intake conditions could guide future research on the genetic basis of fatty liver. The identification of differentially expressed genes, including mRNAs, circRNAs, and lncRNAs, provides a foundation for understanding the molecular underpinnings of energy metabolism and fat deposition in the goose species. Understanding these genetic factors may inform targeted interventions for improved health and breeding strategies, with potential applications in poultry husbandry and comparative genomics.

**Abstract:**

The lion-head goose is the only large goose species in China, and it is one of the largest goose species in the world. Lion-head geese have a strong tolerance for massive energy intake and show a priority of fat accumulation in liver tissue through special feeding. Therefore, the aim of this study was to investigate the impact of high feed intake compared to normal feeding conditions on the transcriptome changes associated with fatty liver development in lion-head geese. In this study, 20 healthy adult lion-head geese were randomly assigned to a control group (CONTROL, n = 10) and high-intake-fed group (CASE, n = 10). After 38 d of treatment, all geese were sacrificed, and liver samples were collected. Three geese were randomly selected from the CONTROL and CASE groups, respectively, to perform whole-transcriptome analysis to analyze the key regulatory genes. We identified 716 differentially expressed mRNAs, 145 differentially expressed circRNAs, and 39 differentially expressed lncRNAs, including upregulated and downregulated genes. GO enrichment analysis showed that these genes were significantly enriched in molecular function. The node degree analysis and centrality metrics of the mRNA–lncRNA–circRNA triple regulatory network indicate the presence of crucial functional nodes in the network. We identified differentially expressed genes, including *HSPB9*, *Pgk1*, *Hsp70*, *ME2*, malic enzyme, *HSP90*, *FADS1*, transferrin, *FABP*, *PKM2*, *Serpin2*, and *PKS*, and we additionally confirmed the accuracy of sequencing at the RNA level. In this study, we studied for the first time the important differential genes that regulate fatty liver in high-intake feeding of the lion-head goose. In summary, these differentially expressed genes may play important roles in fatty liver development in the lion-head goose, and the functions and mechanisms should be investigated in future studies.

## 1. Introduction

Non-alcoholic fatty liver disease (NAFLD) is one of the most common chronic liver diseases, with a prevalence rate of around 25% globally [1]. A study spanning nearly 30 years has showed that the disease burden of NAFLD in the Chinese population has been rising significantly in recent years and is associated with multiple risk factors [2,3]. Research on fatty liver mainly consists of observing the clinical characteristics of mice after specific gene editing or studying the safety and efficacy of specific drugs for patients with NAFLD. Currently, clinical research on fatty liver patients is limited to mild symptoms and late-stage studies and, because of the complex, multidirectional pathophysiology involved in NAFLD, the perfect animal model representing the complete NAFLD spectrum in a workable time frame does not exist [4,5]. Interestingly, geese have a special fat deposition pattern and have a very high capacity for accumulating fat, which can cause the size of the liver to increase several times after overfeeding with a high-energy diet rich in carbohydrates. This distinctive genetic characteristic of the goose has been taken advantage of to produce foie gras. Research suggests that the fatty livers of geese produced by overfeeding have similar characteristics to human non-alcoholic fatty liver, and their serum enzyme levels are close to those of patients with NAFLD [6]. Other studies have shown that compared to other mammals, the inflammatory reaction and endoplasmic reticulum stress of geese are suppressed to some extent, indicating that geese may have protection for the liver and prevent serious diseases [7]. Therefore, through artificially controlling the filling feed, the growth of the liver can be controlled, which has good potential for research into the severity of fatty liver and as a model for human NAFLD [8].

At the same time, the nutritional value of fattened goose liver is very high. With the improvement of people’s living standards, the demand for fattened goose liver will gradually increase. The variety of goose is an important factor affecting fatty goose liver. Among different breeds of geese, lion-head geese, due to their large size and strong digestive capacity, are very conducive to feeding and producing liver, making them one of the most ideal varieties for producing fatty goose liver [9,10,11]. France is the world’s largest producer of foie gras. During the fattening process, breeders use artificial methods to insert a 15-cm-long tube into the goose’s esophagus, repeatedly filling it with feed and force-feeding, which rapidly increases the liver’s weight, size, and fat content. The production of foie gras, especially the force-feeding method, has long been a focal point of animal welfare controversy. This practice has been legally restricted or banned in some countries and regions. As public awareness of animal welfare increases and technology advances, the foie gras industry may continue to develop, and it is necessary to continuously improve production methods to reduce the negative impact on animals.

The ancestor of lion-head goose is the lion-head wild goose, which is the world’s largest goose species with the beautiful name of “World Goose King” [12,13]. It has a large head and tubercles so it is called the lion-head goose. At present, the main producing area of the lion-head goose is Chenghai District, Shantou City, Guangdong Province. After continuous selection and breeding, the current lion-head goose has the characteristics of fast growth, rough feeding, strong adaptability, and low environmental requirements [14]. The adult domestic geese from similar regions vary greatly in weight and size. The lion-head goose in Shantou, Guangdong Province, has a large body, a deep and wide head, and five large sarcomas on the front and side of the face [15]. The adult weight of the lion-head goose is 9~10 kg for males and 7.5~9 kg for females, and it grows rapidly and has rich muscles [16]. At present, there is no relevant research on the fatty liver of the lion-head goose. Therefore, the research on the fatty liver of the lion-head goose can not only find the key regulatory gene for the fatty liver of the lion-head goose when preserving the original excellent traits from breeding but also can effectively improve its economic benefits. The lion-head goose can become a good research model for different degrees of fatty liver in humans, providing a reference for the study of fat metabolism disorders in humans.

In this study, the lion-head geese populations in Shantou were selected for whole-transcriptome sequencing from their liver tissues. First, 716 differentially expressed mRNAs of liver between high-intake and normal feeding were identified by RNA-seq. Second, the relative expression differences between high-intake and normally fed geese of different mRNAs were analyzed by qRT-PCR. *HSPB9*, *Pgk1*, *Hsp70*, *ME2*, malic enzyme, *HSP90*, and *FADS1* were highly expressed in geese with high intake. Third, the critical lncRNA–mRNA–circRNA regulatory network of livers was constructed in the high-intake-fed and normally fed lion-head geese. This will help us to understand the molecular mechanism of fatty liver development in lion-head geese from transcriptomic perspectives.

## 2. Materials and Methods

### 2.1. Animal Samples

The lion-head geese were treated in accordance with the South China Agricultural University Committee of Animal Experiments and this was approved by the bioethics committee of ULPGC (SYXK2019-0136).

### 2.2. Animal Handling and Sample Collection

Twenty healthy adult lion-head geese at 120 days of age, which were purebred, were obtained from the Shantou Baisha Research Institute of Original Species of Poultry and Stock. Ten lion-head geese were randomly selected as the high-intake-fed group (labeled as CASE), while another ten lion-head geese were selected as the control group (labeled as CONTROL). The lion-head geese chosen for this study were raised in enclosed enclosures under the same environmental conditions, with each group in a separate dedicated isolator. After more than 10 generations of selective breeding, the lion-head geese involved in this study belonged to the same original population, with consistent body size and health status. All of the lion-head geese had free access to water at all times [17]. All of the geese were provided with the same diet consisting of 10% crude protein, 12% coarse fiber, 0.33~1% calcium, 0.25~0.8% water-soluble oxide, 0.55% lysine, 1.5% crude fat, 12% crude ash, 0.3~0.9% methionine, and 0.2% total phosphorus. However, the two groups differed in terms of daily feeding times. The control group had free access to the diet throughout the day and consumed an average of 356 g (energy level 5.08 MJ/Kg) of feed per day. The feeding procedure for the high-intake-fed group was similar to what is used in the industry to produce foie gras. The diet included rice boiled for 60 min and more. The birds were artificially force-fed twice a day for days 1~3, three times a day for days 4~6, four times a day for days 7~25, and three times on day 26. The meals were 200 g for days 1~3. The meals on day 4 were 300 g and fed every day until the end. On day 38, the day before the birds were sacrificed, all geese were deprived of feed overnight. There was a difference of 81.44 MJ in energy intake between the two groups for each goose. The geese at the end of the study were euthanized by the intravenous administration of sodium pentobarbital (100 mg/kg body weight). The body weight of each goose was recorded prior to euthanasia, including 10 female and 10 male lion-head geese. Liver weight and size were also recorded for each goose after euthanasia. 

### 2.3. Total RNA-Seq and Quality Control

Liver sample extracts from high-intake-fed lion-head geese and normally fed lion-head geese were randomly selected to make the libraries (3 biorepeats per group), and each library originated from the RNA of a single individual. Total RNA was extracted using the TRIzol reagent kit (Invitrogen, Carlsbad, CA, USA) in accordance with the manufacturer’s protocol. The concentration and purity of RNA were measured using the NanoDrop 2000 spectrophotometer (Thermo Fisher Scientific, Wilmington, DE, USA), based on the OD ratio of 260/280, while the RNA concentration was quantified by fluorescence spectroscopy (Qubit 2.0) [18]. Subsequently, RNA integrity was evaluated using the Bioanalyzer 2100 system (Agilent Technologies, Santa Clara, CA, USA) [19]. Samples with an average RNA integrity number (RIN) value of 7.59 (ranging from 7 to 8.6) were then sent to MingLead Gene (Guangzhou, China) for the generation of paired-end libraries [20]. All libraries were sequenced following Illumina’s protocols by MingLead Gene. MingLead Gene also performed standard quality control and filtering to remove low-quality reads, providing clean reads for subsequent analysis.

### 2.4. Preparing and Constructing cDNA Library

Total RNA was utilized as the input material for the RNA sample preparations. For the lncRNA libraries, mRNA was purified from total RNA using probes to remove rRNA [21]. Fragmentation was carried out using divalent cations under elevated temperature in First Strand Synthesis Reaction Buffer (5×). First-strand cDNA synthesis was performed using a random hexamer primer and M-MuLV Reverse Transcriptase (RNase H) [22]. Subsequently, second-strand cDNA synthesis was carried out using DNA Polymerase I and RNase H [22]. Any remaining overhangs were converted into blunt ends through exonuclease/polymerase activities. Following the adenylation of 3′ ends of DNA fragments, NEBNext Adaptor with a hairpin loop structure was ligated to prepare for hybridization [23]. To selectively isolate cDNA fragments ranging from 370~420 base pairs (bp) in length, library fragments were purified using the AMPure XP system (Beckman Coulter, Beverly, MA, USA). Subsequently, 3 µL of USER Enzyme (NEB, Ipswich, MA, USA) was used with the size-selected, adaptor-ligated cDNA at 37 °C for 15 min, followed by 5 min at 95 °C before PCR amplification. PCR was performed with Phusion High-Fidelity DNA polymerase, universal PCR primers, and Index (X) Primer. Finally, PCR products were purified (AMPure XP system), and the library quality was assessed using the Agilent Fragment Analyzer 5400 system [24]. 

### 2.5. Transcriptome Sequence Data Analysis

For sequencing, the Illumina HiSeqTM 4000 platform was employed, and the samples were sent to MingLead Gene Technology Co., Ltd. (Guangzhou, China). The raw data underwent quality filtering. Initially, reads containing adapters were excluded, followed by the exclusion of reads with N ratio exceeding 10%, reads with A bases, and low-quality reads (where the number of bases with a mass value Q ≤ 20 constituted more than 50% of the whole read) [25]. Subsequently, the short-read alignment tool Bowtie2 was utilized to classify and compare the reads with ribosomes. Unmapped reads were retained for subsequent transcriptome analysis [26]. Comparative analysis based on the goose genome was performed using the HISAT2 software [27]. Sample expression levels were determined using fragments per kilobase of transcript per million fragments mapped (FPKM), and the sample repeatability was assessed through principal component analysis [28,29].

### 2.6. lncRNA and Circular RNA Identification

To identify lncRNAs, quality control is performed using Fastp to ensure that the RNA sequencing data meet specified standards, including Q20 ≥ 40%, N bases <5, and read length ≥50. Subsequently, rRNA is removed from the data using Bowtie2, with RNACentral serving as the rRNA database. Following this, the processed data are aligned to the corresponding reference genome using Hisat2 to determine the position of RNA fragments on the genome [30]. Stringtie is then employed to assemble transcripts from the aligned data, identifying and stitching together all potential transcripts. To integrate transcript information from multiple samples, the merge parameter of Stringtie is applied for consolidation. Gffcompare is utilized to compare the assembled GTF file with the GTF annotation file of the reference genome, assessing the consistency of the assembled transcripts with known annotations. Ultimately, transcripts with potential lncRNA characteristics are identified through further analysis and filtering.

Following this, CIRIquant is utilized for alignment to the reference genome using Hisat2 and gene-level quantification is performed using Stringtie [31]. Simultaneously, circRNA identification is conducted by aligning with the reference genome using bwa-mem, and a circRNA reference sequence is constructed. The constructed circRNA sequence is then employed as the reference genome for another round of alignment with Hisat2, allowing the filtering of high-confidence reads originating from circular RNA. Subsequently, circRNA expression levels are quantified, and circRNA information is annotated. Differential analysis of circRNAs is carried out to select circRNAs with significant differences.

### 2.7. Analysis of Differentially Expressed Genes

Gene expression levels were quantified using FPKM. By comparing the FPKM values of genes, expression differences between the two groups were examined for both genes and transcripts [32]. Screening of differentially expressed gene condition was carried out based on a fold change >2 and a *p*-value <0.05 [33]. The sequencing data from the two groups were compared, and the differentially expressed mRNAs were visualized using the ggplot2 software package to generate volcano plots [34]. These plots illustrated the magnitude of expression differences and the statistical significance, providing an intuitive representation of the gene distribution.

### 2.8. Function Enrichment Analysis of DEGs

Gene Ontology analysis was conducted to assess the differential expression data using the HTseq software [35]. The R package edgeR was employed for the analysis of differential expression [36]. The identification of differentially expressed and target genes was carried out using KOBAS v2.0, employing a hypergeometric test [37,38]. Significantly enriched gene sets were determined based on a *p*-value threshold of <0.05.

### 2.9. Quantitative Real-Time PCR (qRT-PCR) Verification

RNAs were extracted from liver tissue samples of the 3 CASE groups and 3 CONTROL groups for qRT-PCR verification. cDNA synthesis was performed using a Takara reverse transcriptase kit (Takara, Dalian, China) following the manufacturer’s instructions. Subsequently, qRT-PCR was carried out in a 20 μL reaction system comprising 10 μL of SYBR Green Master Mix (Vazyme, Nanjing, China), 0.5 μL of PCR forward primer (10 μM), 0.5 μL of PCR reverse primer (10 μM), 1 μL of cDNA, and 8 μL of enzyme-free water. The reaction system began to react at 95 °C for 3 min and then reacted at 95 °C (5 s) and 60 °C (34 s) for a total of 40 cycles. The reaction was carried out in three separate wells, with GAPDH as an internal reference, and the relative RNA expression level was calculated using the 2−ΔΔCt value [39]. A *t*-test was performed to determine statistical significance, and the results were presented as mean ± standard deviation. For validation, we randomly selected 11 differentially expressed mRNAs from the sequencing group, including 7 upregulated mRNAs (Gene ID: *HSPB9*, *Pgk*, *Hsp70*, *ME2*, malic enzyme, *HSP90*, *FADS1*) and 4 downregulated mRNAs (Gene ID: transferrin, *FABP*, *PKS*, Serpin2). The primer sequences for these 11 genes are provided in Table 1.

### 2.10. Statistical Analysis

GraphPad Prism 8.0 (GraphPad Software, La Jolla, CA, USA) was used for statistical analysis. Paired Student’s *t*-tests were performed between normally fed livers and high-intake-fed livers. The statistical significance was defined as *p* < 0.05.

## 3. Results

### 3.1. Phenotypic Changes in the Liver of Lion-Head Geese Following High-Intake Feeding 

**Figure 1:**
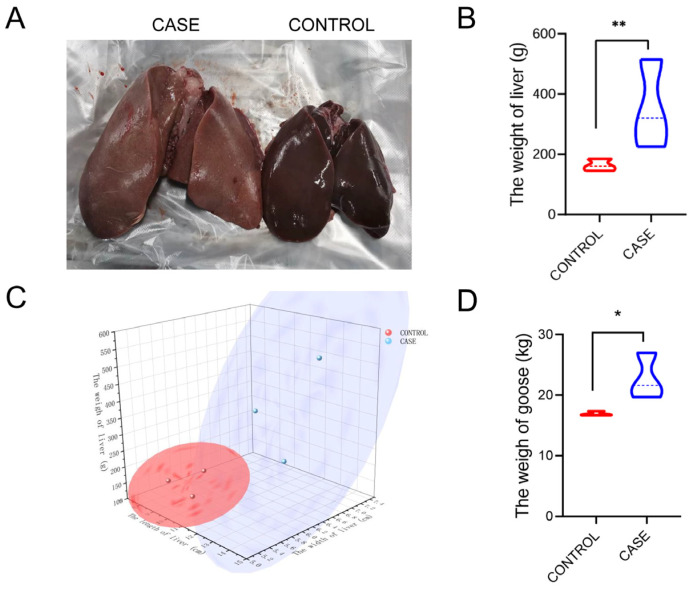
Phenotypic changes in liver and body weight after high-intake feeding in lion-head geese. (**A**) Goose liver in the groups of high-intake feeding and normal feeding. (**B**) The weight of liver. (**C**) The size of the goose liver. (**D**) The weight of goose (** *p* < 0.01; * *p* < 0.05).

To comprehensively analyze the whole-transcriptome of livers in lion-head geese, we collected liver data (10 geese per group) from different treatment groups including high-intake-fed and normally fed lion-head geese at the same time. The liver phenotype, weights, and size, as well as body weight of high-intake-fed lion-head geese, exhibited significant differences compared to those of normally fed lion-head geese at the end of the fattening process (Figure 1A). Tissues associated with lipid metabolism showed increased weight after the fattening period, with the liver exhibiting the highest relative weight increase of 1.8-fold (*p* < 0.01) (Figure 1B), thereby contributing significantly to the overall weight gain in high-intake-fed lion-head geese (*p* < 0.05) (Figure 1D). Additionally, upon assessing the liver and body weight, we observed a substantial increase in liver size of high-intake-fed compared to normally fed lion-head geese (Figure 1C).

### 3.2. Characterization of the Liver Tissue Transcriptome Data in Lion-Head Geese 

We obtained a total of 125,417,714 raw reads for the liver tissue libraries of high-intake-fed lion-head geese (n = 3) and 126,982,352 raw reads for the libraries from normally fed lion-head geese (n = 3) through RNA-seq analysis. After filtering and removal of sequence reads with adapters and low quality, we obtained 39,277,879 and 40,199,239 clean reads for high-intake-fed and normally fed lion-head geese. The rates of clean reads mapped were 93.94% and 94.97%. The Q20 and Q30 values exceeded 97% and 93%, respectively, and the GC content was above 45% [40]. Finally, 71.53 Gb of clean data were obtained, with each sample averaging around 11 Gb. Detailed analysis data of the sequencing samples are presented in Table 2.

### 3.3. Overview of Whole-Transcriptome Sequencing in Lion-Head Geese

**Figure 2:**
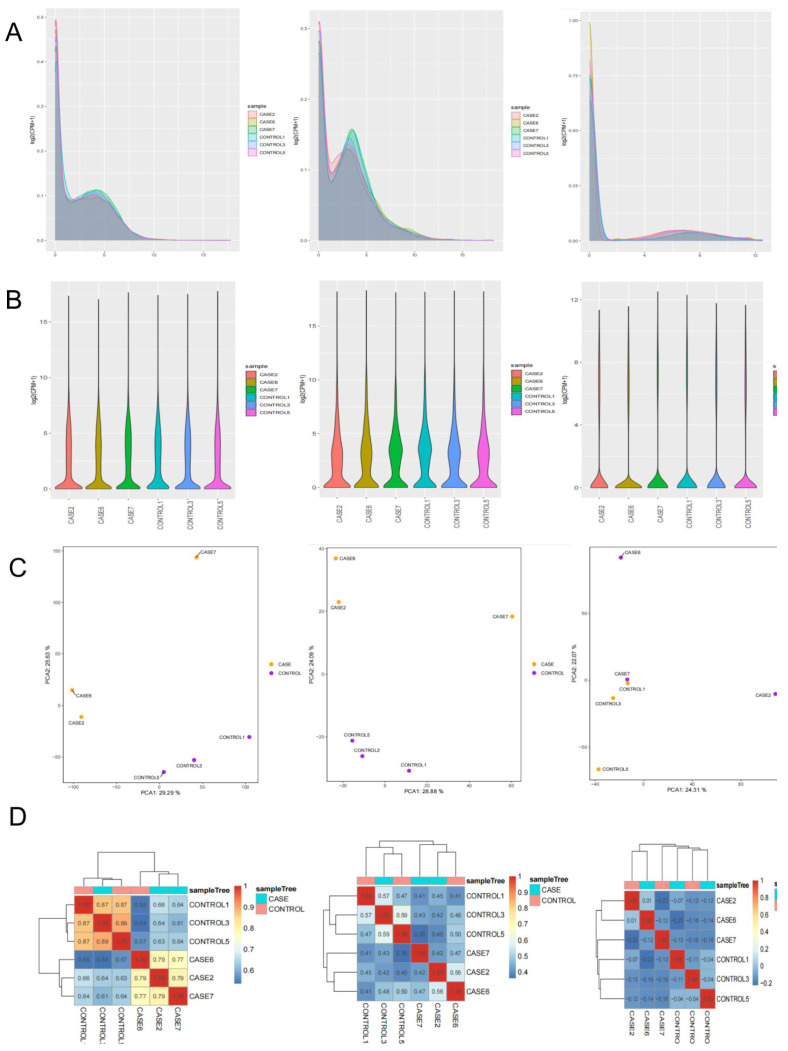
Overview of whole-transcriptome sequencing. (**A**) The density distribution of transcriptome was according to log10 (FPKM), original gene read counts were normalized using the FPKM method. (**B**) The 6−sample expression violin plot. (**C**) The principal component analysis (PCA) is useful for exploring the distance relationship between the 6 samples. (**D**) Pearson’s correlation matrix for mRNA, lncRNA, and circRNA profiles.

To obtain whole and accurate mRNA transcripts of the goose liver, we constructed six cDNA libraries (CASE2, CASE6, CASE7, CONTROL1, CONTROL3, and CONTROL) from liver tissue of lion-head geese. Using RNA-seq, we detected 21,008 mRNA transcripts, 3549 lncRNA transcripts, and 10,284 circRNA transcripts. Transcript expression was quantified using FPKM value [41]. The FPKM distribution of mRNAs is shown in Figure 2A, while the expression of different samples is displayed as a violin chart (Figure 2B). To effectively identify the most significant element and structures in the data, we represented the complex composition relationship of samples using horizontal and vertical coordinates [42]. This approach facilitated the exploration of the distance relationship between samples. The six samples were divided into two groups, which showed satisfactory repeatability (Figure 2C). Furthermore, we generated a hierarchical clustering diagram to visually depict the relationships between samples. Unsupervised clustering also confirmed the distinct expression patterns among liver tissues (Figure 2D). Sequences showed a reliable clustering effect, which ensured the veracity of the subsequent analysis [43].

### 3.4. Differential Expression of mRNA, lncRNA, and circRNA between Liver Samples from High-Intake-Fed and Normally Fed Lion-Head Geese 

**Figure 3:**
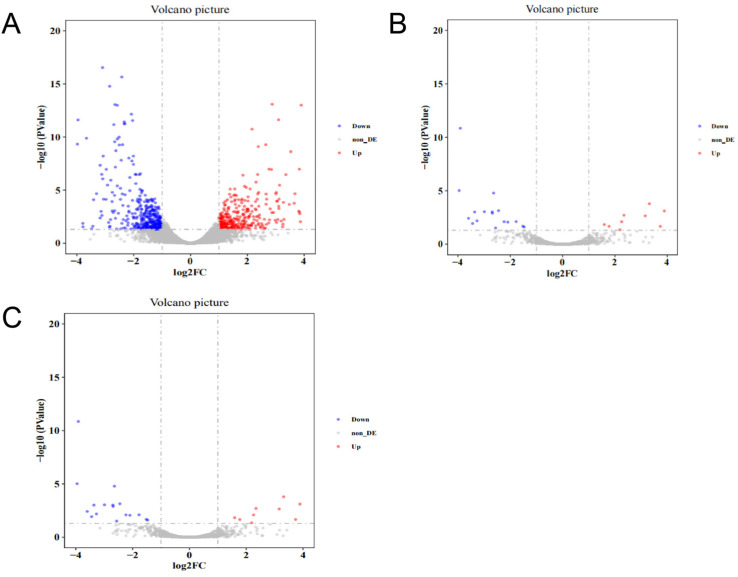
Identification and analysis of mRNA, lncRNA, and circRNA associated with fatty liver. (**A**) Volcano plots of DEGs in mRNA. (**B**) Volcano plots of DEGs in lncRNA. (**C**) Volcano plots of DEGs in circRNA. Each dot in the plot represents a gene with its corresponding log2 fold change (FC) on the x-axis and *p*-value (log10) on the y-axis. The horizontal line indicates the significance threshold (false discovery rate <5%), whereas the vertical line segregates genes with logFC >1.5. Gray represents no significant difference between the two groups, red indicates the upregulated genes, and blue indicates the downregulated genes.

A total of 716 differentially expressed mRNAs, 39 differentially expressed lncRNAs, and 145 differentially expressed circRNAs were identified in livers by comparing sequencing results between high-intake-fed lion-head geese and normally fed lion-head geese. Among these, 335 mRNAs were upregulated and 381 mRNAs were downregulated in the group of high-intake-fed lion-head geese compared to the group of normally fed lion-head geese (Figure 3A). Additionally, 16 lncRNAs were upregulated and 23 lncRNAs were downregulated (Figure 3B), while 91 circRNAs were upregulated and 54 circRNAs were downregulated in livers between the group of high-intake-fed lion-head geese and the group of normally fed lion-head geese (Figure 3C). These results indicate a significant difference in the transcriptome level between high-intake-fed lion-head geese and normally fed lion-head geese in the volcano plot, in which each point represents a transcript, with the x-axis representing the log2 fold change in transcript expression between the two groups and the y-axis representing the negative logarithm of the *p*-value associated with the transcript changes. A larger absolute value on the x-axis indicates a greater difference in expression between the two groups, while a higher y-axis value indicates a more significant difference. 

### 3.5. Functional Enrichment by GO Analysis 

**Figure 4:**
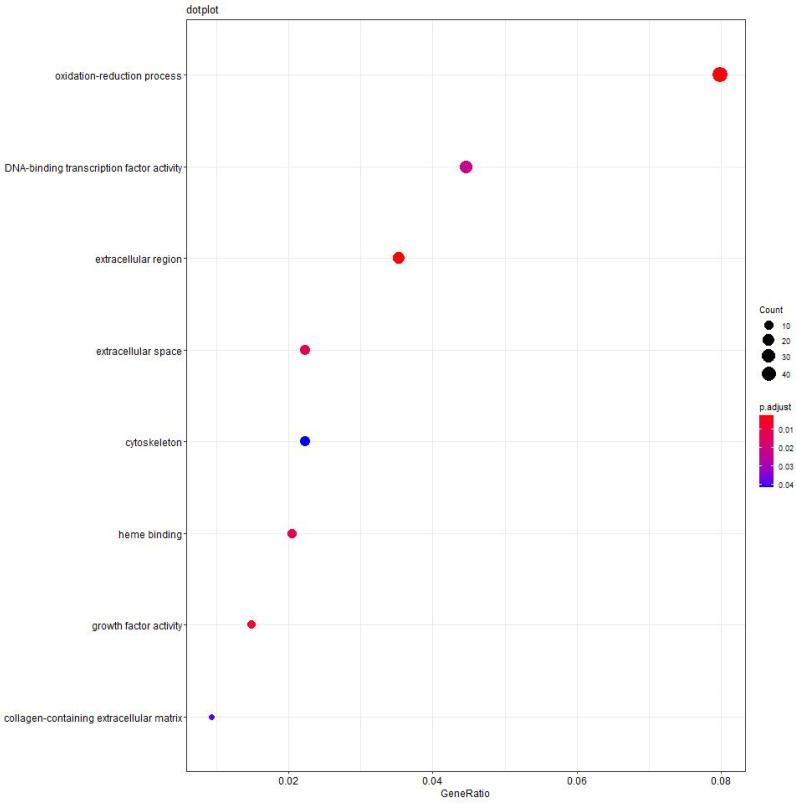
Functional analyses of significantly enriched trends. The top 8 significant terms at the mRNA level. The size of dots shows the number of DEGs clustered in the same terms. The color of dots indicates the *p*-value.

To gain a better understanding of the functions of these DEGs, we performed additional Gene Ontology (GO) analyses between high-intake-fed lion-head geese and normally fed lion-head geese [44]. The KOBAS tools were used to conduct GO enrichment analysis for the DEGs [45]. Our analysis revealed a significant enrichment of DEGs in the oxidation–reduction process. In brief, we initially identified the DEGs, followed by a functional enrichment analysis of these genes in the liver of high-intake-fed lion-head geese. Notably, the most enriched GO terms were oxidation–reduction process (GO.0005576) and DNA-binding transcription factor activity (GO.0003700). We employed the same methodology to predict the functions of the lncRNAs and circRNAs. The top three enrichment terms of the DEGs are oxidation–reduction process, DNA-binding transcription factor activity, and extracellular region. The top eight enrichment terms are presented in Figure 4, including oxidation–reduction process, DNA-binding transcription factor activity, extracellular region, extracellular space, heme binding, growth factor activity, and collagen-containing extracellular matrix. 

### 3.6. Construction and Visualization of lncRNA–mRNA–circRNA Network 

**Figure 5:**
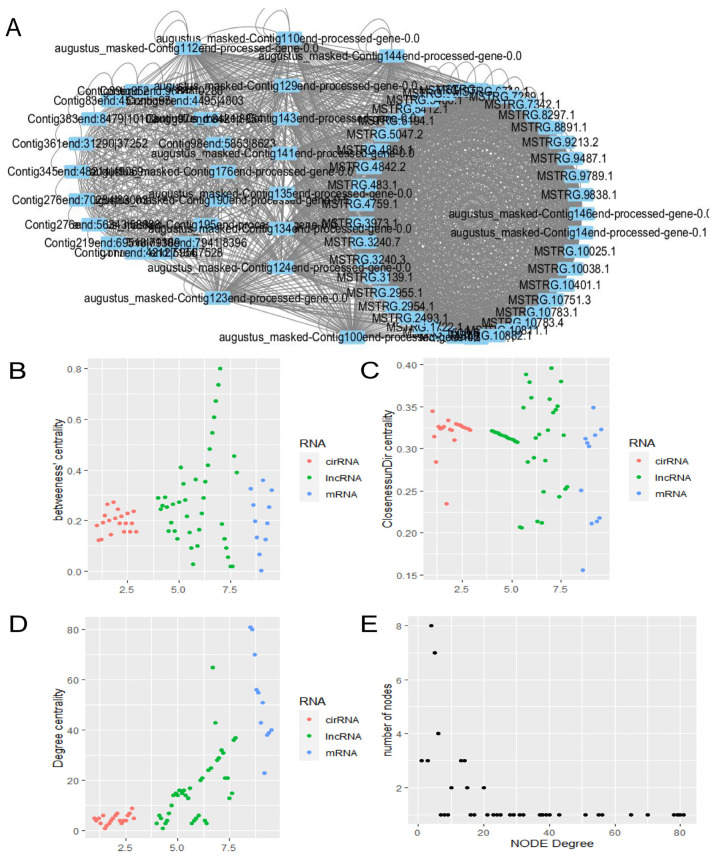
Construction and visualization of lncRNA–mRNA–circRNA network. (**A**) lncRNA–mRNA–circRNA network structure. (**B**) The betweenness centrality difference among lncRNAs, mRNAs, and circRNAs. (**C**) The closeness centrality difference among lncRNAs, mRNAs, and circRNAs. (**D**) The degree centrality difference among lncRNAs, mRNAs, and circRNAs. (**E**) Node degree of the lncRNAs, mRNAs, and circRNAs.

Subsequently, we constructed a critical lncRNA–mRNA–circRNA regulatory network of livers in the high-intake-fed and normally fed lion-head geese (Figure 5). In Figure 5A, a triple regulatory network involving mRNA–lncRNA–circRNA is illustrated. The augustus node represents mRNA, the Contig node represents circRNA, and the MSTRG node represents lncRNA. From the network, it is evident that there is a close interconnection among different RNAs, indicating the presence of intricate regulatory relationships among the three. The x-axis of the betweenness centrality graph represents RNA types, while the y-axis represents the betweenness centrality values corresponding to various RNAs. Betweenness centrality measures a node’s ability to act as an intermediary in the network. In Figure 5B, it is observed that lncRNAs exhibit relatively high betweenness centrality, indicating that these lncRNAs may play crucial bridging roles in the network, possessing key regulatory functions.

The closeness centrality graph illustrates the closeness centrality measure, which assesses the average distance of a node to other nodes in the network. In Figure 5C, it is observed that the closeness centrality of the three RNA types is relatively high, with no significant differences. This suggests the potential presence of close interactions and regulatory relationships among the three RNA types. Figure 5D represents the degree centrality graph, where degree centrality measures the number of direct connections a node has with other nodes. In the graph, mRNA exhibits the highest average degree centrality, followed by lncRNA, and circRNA shows the lowest. This indicates that mRNA may have a greater regulatory potential in the network. And Figure 5E presents the results of the node degree analysis of the network. The x-axis represents the degree of nodes, while the y-axis represents the number of nodes. The results conform to a power-law distribution, indicating that the majority of “ordinary” nodes have few connections, while a minority of “hub” nodes have a significantly higher number of connections. This observation suggests that the network exhibits typical characteristics of a biological scale-free network. In general, the presence of a scale-free distribution and modular characteristics in this network indicates the existence of functional nodes associated with fatty liver in lion-head geese.

### 3.7. Validation of Candidate Genes by qRT-PCR

**Figure 6:**
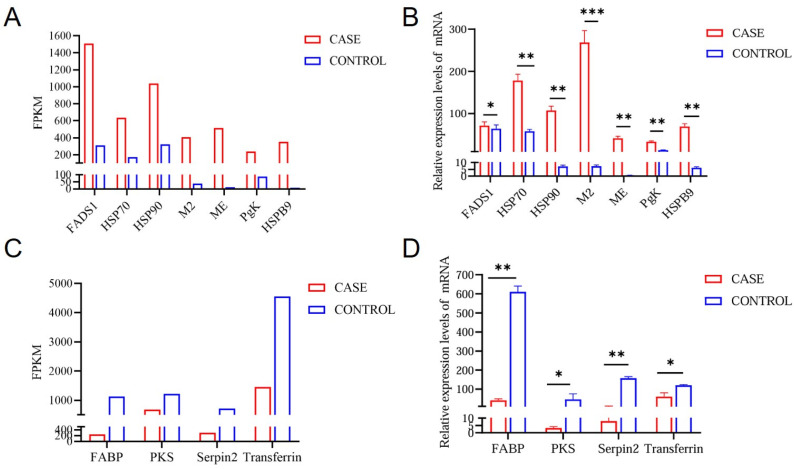
Comparison and identification of differentially expressed RNAs in high-intake feeding in lion-head geese liver tissue by qRT-PCR. (**A**) The RNA-seq results revealed the upregulated genes by FPKM. (**B**) Verification of the upregulated genes by RT-qPCR. (**C**) The RNA-seq results revealed the downregulated genes by FPKM. (**D**) Verification of the downregulated genes by RT-qPCR. Log2 FC is expressed as mean ± SD. n = 3. The statistical significance of all genes reached *p* < 0.05 (*** *p* < 0.001; ** *p* < 0.01; * *p* < 0.05).

To validate the consistency of mRNA expression levels with the sequencing analysis data, we randomly selected seven upregulated genes and four downregulated genes from liver tissue for qRT-PCR. Based on the FPKM value, we observed significantly higher expression levels of *FADS1*, *HSP70*, *HSP90*, *M2*, *ME*, *PgK*, and *HSPB9* in high-intake-fed lion-head geese, whereas the expression levels of FABP, PKS, Serpin2, and transferrin in liver tissues were significantly lower than those in normally fed lion-head geese (Figure 6B,D). The expression trend of the mRNA, as determined by qRT-PCR, was consistent with those observed in the RNA-seq based on FPKM values (Figure 6A,C), partially supporting the high reliability of our RNA-seq data.

## 4. Discussion

Foie gras, cherished for its unique texture and rich fat content, is a beloved delicacy and a culinary choice for many. It is abundant in high-quality fats, primarily monounsaturated fatty acids, which contribute to its status as a high-energy food source [46,47]. Fats play a significant role in foie gras, primarily deriving from the liver’s metabolic processes [48]. Geese are fed substantial amounts of feed, which contains a high proportion of carbohydrates and other energy, stimulating the synthesis and storage of fat in the liver cells to artificially increase the fat content [49,50]. The large amounts of feed are artificially introduced directly into the geese’s stomachs to ensure they receive adequate energy. Due to this high energy intake, the liver cells of the geese accelerate the synthesis and storage of fat, leading to a significant enlargement of the liver size.

In this study, we conducted transcriptome analysis of gene regulatory networks to compare the phenotypic changes in liver development between the high-intake-fed lion-head goose and the normally fed goose. Simply put, we compared the differences in lncRNA, circRNA, and mRNA expression in the livers of the two groups of geese, further elucidating the molecular regulation that influences lipid accumulation in the liver of the lion-head goose. Comparing the growth and hepatic fat traits between high-intake-fed and normally fed lion-head geese will help us understand two distinct phenotypes with similar genetic backgrounds [51]. Next, we identified differentially expressed genes in the liver tissue transcriptomes of the high-intake-fed and normally fed lion-head geese. Our analysis revealed 716 differentially expressed mRNAs, 145 differentially expressed circRNAs, and 39 differentially expressed lncRNAs in livers, demonstrating significant expression differences between the high-intake-fed and normally fed lion-head geese. Specifically, compared to the normally fed geese, the liver tissue of the high-intake-fed lion-head geese exhibited upregulation of 335 mRNA genes and downregulation of 381 mRNA genes. These results suggest that these differentially expressed genes may play crucial roles in determining the developmental differences in the liver between the two groups of geese.

Furthermore, we conducted the functional enrichment analysis to study the significant differences between high-intake-fed and normally fed lion-head geese. GO enrichment analysis revealed significant enrichment in the oxidative–reduction processes and DNA-binding transcription factor activity. It has been suggested that mitochondrial oxidative function plays a crucial role in the development of NAFLD, and hepatic steatosis may not be a result of reduced mitochondrial fatty acid oxidation caused by mitochondrial damage [52,53]. In this process, NAFLD can induce an elevation in mitochondrial function, compensating for the increased demand for carbon intermediates and ATP caused by elevated lipogenesis and gluconeogenesis [54]. The liver is a vital metabolic organ responsible for the synthesis, breakdown, and regulation of various biomolecules, primarily achieved through gene transcription [55,56]. The liver contains numerous specific DNA-binding transcription factors, such as liver X receptors (LXRs), hepatocyte nuclear factors (HNFs), and progesterone receptors [57]. These factors can bind to specific DNA sequences in the promoter regions, recruiting co-activators or co-repressors to regulate the initiation and rate of gene transcription [58]. The specificity of the transcription factors binding to DNA provides a mechanism for precise regulation of gene expression in liver cells, thereby enabling control and adaptation of metabolic processes.

Next, we selected a series of upregulated and downregulated mRNA to validate the reliability and accuracy of the sequencing data. Heat shock proteins (HSPs) are a class of widely expressed proteins in cells that play a vital role in protecting cells from damage and promoting cellular recovery and repair processes under various stress conditions [59]. HSPs play an important protective role in the liver of animals, particularly in the regulation of intracellular lipid metabolism and oxidative stress [60]. This can also scientifically explain why overfeeding can lead to upregulation of HSP expression in the lion-head goose. An upregulated expression can be considered as an adaptive response of liver cells to stressful conditions, aiming to protect liver cells from damage and maintain their function. PgK is one of the key enzymes in the glycolytic pathway and participates in glycolysis [61]. After high-intake feeding, the glycolytic pathway in the liver may be affected, leading to changes in the activity or expression levels of PgK in liver tissue, thereby influencing lipid metabolism. Malic enzyme, a crucial enzyme in cellular energy and lipid metabolism, plays a pivotal role in the metabolism of pyruvate and the generation of nicotinamide adenine dinucleotide phosphate (NADPH) [62]. Specifically, it catalyzes the conversion of malate to pyruvate, simultaneously producing NADPH from NADP. Research indicates that in instances of liver lipid accumulation disrupting hepatic lipid metabolism, there is a significant increase in the activity or expression levels of malic enzyme. This underscores the enzyme’s vital contribution as a primary provider of NADPH, essential for lipogenesis [63]. 

In the sequencing data of the lion-head goose, we simultaneously validated the expression of a subset of downregulated genes. Real-time quantitative PCR results showed that the expression trends of differentially expressed RNAs were consistent with the whole-transcriptome sequencing data. Hepatic steatosis is often accompanied by increased inflammation and oxidative stress, indicating an imbalance in intracellular redox homeostasis [64,65]. Serpin2 possesses anti-inflammatory and antioxidant properties [66], and the expression levels of Serpin2 may be negatively regulated due to the interplay between inflammatory responses and disrupted lipid metabolism. FABPs belong to the lipocalin family and participate in the transport and metabolism of fatty acids [67]. In the case of hepatic lipid accumulation, abnormal expression or functional defects of FABPs may disrupt intracellular lipid metabolism. Studies have indicated that FABPs are involved in regulating inflammation-related signaling pathways and cytokine expression, thereby affecting the severity of inflammatory responses and the progression of liver fibrosis [68]. Although there is a lack of direct research evidence on the relationship between fatty liver and PKS, we validated that the expression of PKS in the liver of lion-head geese was lower than in the liver of normally fed geese. Some studies suggest that PKS is involved in the synthesis of polyketide compounds, and certain polyketides may play a role in regulating lipid metabolism [69,70,71]. Further research is needed to confirm and explore the specific relationship between fatty liver and PKS. The verification of the selected genes will help us to understand the molecular mechanism of high-intake lion-headed goose liver from the perspective of the transcriptome.

## 5. Conclusions

We utilized RNA-seq analysis to compare transcriptome of liver tissues between high-intake-fed and normally fed lion-head geese. The expression of these differential genes causes weight, size, and mass differences of liver observed between the high-intake-fed lion-head goose and the normal goose. Meanwhile, we constructed the critical lncRNA–mRNA–circRNA regulatory network of livers in the high-intake-fed and normally fed lion-head geese. Conducting transcriptome analysis of the livers of lion-head geese in Shantou is essential for gaining insights into gene expression regulation, liver development, and cell function. This approach will aid in understanding the molecular mechanisms behind fatty liver development in lion-head geese from a transcriptomic perspective. In the future, comparative studies of liver transcriptomes across different goose species will be instrumental in revealing the variations in liver tissue characteristics among these species.

## Figures and Tables

**Table 1:** The primer sequences of gene for RT-qPCR.

Gene ID	Regulation	Forward Primer 5′→3′	Reverse Primer 5′→3′
Transferrin	Down	GCCTTATTCTGGATATTCTG	CTCATACTCGTCCTTCTC
*FABP*	Down	GAGATATTAAGCCTGTTGTTG	TTGCCGTCCTAGTAGTA
*PKS*	Down	AAGAGGAGAAGCAATATC	CTGTGATGATGTAGACT
Serpin2	Down	GAAGAGGAGGAGGAAGAG	GAGCAGCGTCATAATGT
*HSPB9*	Up	AGGAAGGTGGTGCTGGTG	CTCGTACTTGTAGAAGG
*Pgk*	Up	CATTATTGGTGGTGGGATACA	GTGCTGACATGGCTAACTT
*Hsp70*	Up	TACCTCAGATTGAAGTAACCT	CTTGCCAGTGCTCTTATC
*ME2*	Up	CAACTGCTGAGGTAATAG	GTGCTGAATTGTGACTAA
Malic enzyme	Up	ATAGCAAACCTCATTGTCAT	CGAGTCAACCATCCATATC
*HSP90*	Up	GCATTCTCAGTTCATTGG	TTCTTCAGCCTCATCATC
*FADS1*	Up	CCTGGTACTTCTGGAATGA	TTGAGCCCTATGGTGTAG
*GAPDH*	/	GGTGGTGCTAAGCGTGTCAT	CCCTCCACAATGCCAAAGTT

**Table 2:** Summary of read mapping of the sample genome.

Groups	CONTROL1	CONTROL3	CONTROL5	CASE2	CASE6	CASE7
Raw Reads	41,742,960	43,317,885	41,921,507	40,449,692	41,793,874	43,174,148
Clean Reads	39,528,644	41,176,548	39,892,524	37,758,464	39,236,814	40,838,358
Raw Base (G)	12.52	13.0	12.58	12.13	12.54	12.95
Clean Base (G)	11.86	12.35	11.97	11.33	11.77	12.25
Effective Rate (%)	94.70	95.06	95.16	93.35	93.88	94.59
Error Rate (%)	0.03	0.03	0.03	0.03	0.03	0.03
Q20 (%)	97.77	97.84	97.67	97.79	97.69	97.77
Q30 (%)	93.90	94.06	93.59	93.97	93.78	93.94
GC Content (%)	45.99	46.62	45.69	47.66	48.03	46.73

## Data Availability

All data generated or analyzed during this study were deposited in the Gene Expression Omnibus (GEO) under accession number GSE243829.
